# The Influence of Single Nucleotide Polymorphisms on Vitamin D Receptor Protein Levels and Function in Chronic Liver Disease

**DOI:** 10.3390/ijms241411404

**Published:** 2023-07-13

**Authors:** Evanthia Tourkochristou, Efthymios P. Tsounis, Haralampos Tzoupis, Ioanna Aggeletopoulou, Aggeliki Tsintoni, Theoni Lourida, Georgia Diamantopoulou, Konstantinos Zisimopoulos, Theodora Kafentzi, Anne-Lise de Lastic, Maria Rodi, Theodore Tselios, Konstantinos Thomopoulos, Athanasia Mouzaki, Christos Triantos

**Affiliations:** 1 Division of Gastroenterology, Department of Internal Medicine, Medical School, University of Patras, University Hospital of Patras, 26504 Patras, Greeceiaggel@hotmail.com (I.A.); agtsintoni@gmail.com (A.T.);; 2 Division of Hematology, Department of Internal Medicine, Medical School, University of Patras, 26504 Patras, Greece; 3 Department of Chemistry, University of Patras, 26504 Patras, Greece

**Keywords:** VDR, protein, SNPs, gene expression, liver cirrhosis, HCV

## Abstract

Single nucleotide polymorphisms (SNPs) in the vitamin D receptor (VDR) gene have been associated with chronic liver disease. We investigated the role of VDR SNPs on VDR protein levels and function in patients with chronic liver disease. VDR expression levels were determined in peripheral T lymphocytes (CD3+VDR+), monocytes (CD14+VDR+), and plasma from patients (*n* = 66) and healthy controls (*n* = 38). Genotyping of SNPs and the determination of expression of VDR/vitamin D-related genes were performed by using qPCR. The effect of FokI SNP on vitamin D-binding to VDR was investigated by molecular dynamics simulations. CD14+VDR+ cells were correlated with the MELD score. The ApaI SNP was associated with decreased CD3+VDR+ levels in cirrhotic patients and with higher liver stiffness in HCV patients. The BsmI and TaqI SNPs were associated with increased VDR plasma concentrations in cirrhotic patients and decreased CD14+VDR+ levels in HCV patients. The FokI SNP was associated with increased CD3+VDR+ levels in cirrhotic patients and controls. VDR polymorphisms were significantly related to the expression of genes critical for normal hepatocyte function and immune homeostasis. VDR expression levels were related to the clinical severity of liver disease. VDR SNPs may be related to the progression of chronic liver disease by affecting VDR expression levels.

## 1. Introduction

**Figure 1:**
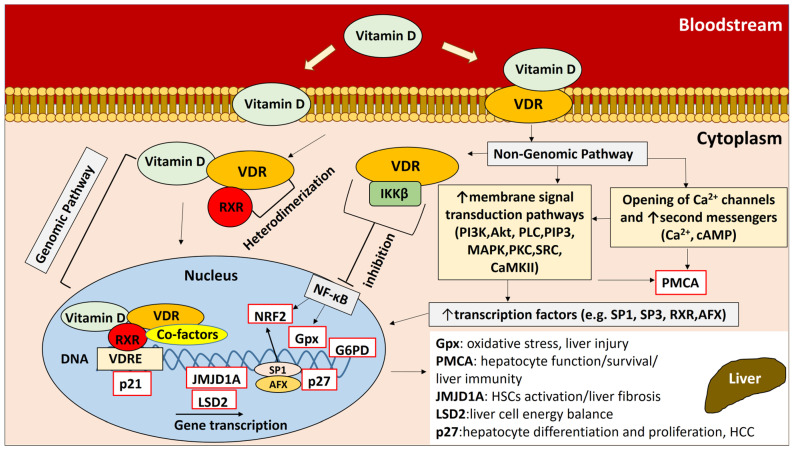
Vitamin D-VDR pathways and regulation of downstream gene expression. Vitamin D exerts its biological effects by interacting with the VDR receptor through two distinct pathways: the genomic pathway and the nongenomic pathway. These pathways play a critical role in regulating downstream gene expression, which can affect hepatocyte and liver tissue homeostasis. (For a detailed description of the vitamin D-VDR pathways and the regulation of downstream gene expression, see the Supplementary Material (Section S1).

Chronic liver disease (CLD) is an important public health issue, as the number of CLD cases worldwide is estimated to be nearly 1.5 billion [1]. Hepatitis C is a liver infection caused by the hepatitis C virus (HCV) and can be a major cause of chronic liver disease, as it can promote severe complications such as cirrhosis, hepatocellular carcinoma, and death [2]. Chronic inflammation in HCV infection gradually leads to long-term tissue damage, which triggers a wound-healing response that results in fibrogenesis and the scarring of liver tissue, a pathological condition known as liver cirrhosis [3]. Liver cirrhosis is the final stage of CLD, and its prevalence continues to increase significantly in recent decades, affecting mortality worldwide [4]. Vitamin D has shown immunomodulatory and antifibrotic effects on the liver [5]. The hormonally active form of vitamin D (1,25(OH)2D3) exerts its pleiotropic effects by binding to the vitamin D receptor (VDR), a transcription factor belonging to the nuclear hormone receptor superfamily [6,7,8,9,10,11,12,13,14,15,16,17,18,19,20,21,22,23,24,25,26] (Figure 1). The human liver is a vitamin D-responsive organ, as VDR is expressed in nonparenchymal liver cells, including Kupffer cells (KCs), sinusoidal endothelial cells, and hepatic stellate cells (HSCs) [27]. Activation of VDR in liver macrophages has been shown to alleviate liver inflammation in chronic liver disease [28]. Vitamin D-VDR signaling may also reduce the development and severity of liver diseases [29,30], including HBV and HCV infections, autoimmune hepatitis (AIH), nonalcoholic fatty liver disease (NAFLD), liver cirrhosis, primary biliary cirrhosis (PBC), and hepatocellular carcinoma (HCC), by regulating signaling pathways that control the expression of anti-inflammatory, anti-proliferative and antifibrotic genes [5,31].

Single nucleotide polymorphisms (SNPs) in the VDR gene have been associated with liver diseases, including AIH, PBC, HBV infection, and HCC [32,33,34,35], and with the rapid progression of fibrosis in patients with HCV infection, PBC, and NAFLD [33,36,37]. Four of the most frequently identified SNPs in the VDR gene are ApaI, BsmI, TaqI, and FokI. The ApaI, BsmI, and TaqI polymorphisms were associated with higher severity of liver cirrhosis, as patients with BsmI and TaqI SNPs had a higher model for end-stage liver disease (MELD) score and were mainly in Child–Pugh (CP) stage C. ApaI SNP was also associated with increased levels of lipopolysaccharide-binding protein (LBP), IL-1β, IL-8, and CP stage C, whereas the CC genotype of the FokI SNP was associated with lower levels of IL-1β and was an independent prognostic factor for the survival of patients with cirrhosis. It has been suggested that the ApaI, BsmI, and TaqI SNPs are associated with the severity of cirrhosis via immunoregulatory processes [38]. Considering the anti-inflammatory and antifibrotic roles of VDR signaling in CLD, the determination of changes in VDR expression and activity associated with specific SNPs in two associated liver diseases, such as HCV and cirrhosis, may indicate the biological mechanism behind the association between VDR SNPs and CLD. 

In this study, we investigated the role of VDR SNPs ApaI, BsmI, TaqI, and FokI in relation to VDR protein levels and function in patients with chronic liver disease, including cirrhosis and hepatitis C virus infection, as well as possible associations between VDR SNPs and clinical characteristics of patients. Elucidating the biological effect of VDR SNPs in CLD will contribute to more efficient management of CLD patients at different stages of liver disease, especially if certain SNPs are found to be associated with a pathogenic or beneficial effect on CLD in the context of personalized medicine.

## 2. Results

The workflow of the samples in each subgroup and the analyses are shown in the figure in Section 4.1. The main demographic and clinical characteristics of the subjects finally included in the study are shown in Table 1, and the characterization and distribution of VDR polymorphisms among the subjects are shown in Table 2. The three groups of subjects from whom biological samples were obtained for all parts of the study were (1) 38 healthy controls, (2) 38 patients with liver cirrhosis, and (3) 28 HCV patients without liver cirrhosis.

### 2.1. VDR Expression Levels in Specific Cell Subsets and Plasma from Healthy Controls Compared with HCV+ and Cirrhotic Patients

**Figure 2:**
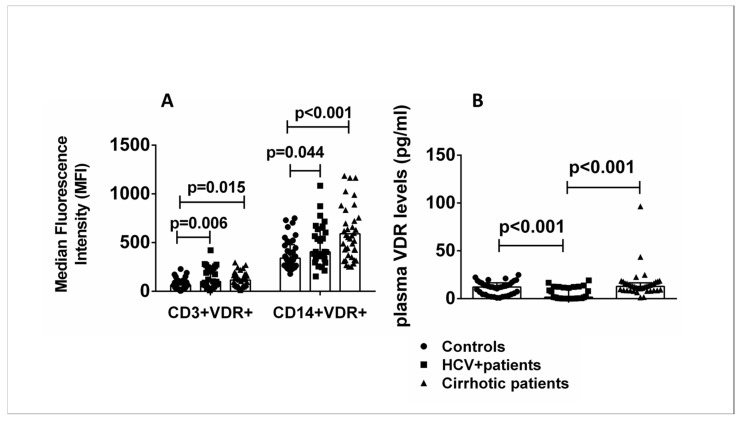
(**A**) CD3+VDR+ and CD14+VDR+ cells in peripheral blood. Results of flow cytometric analysis of all patients and controls. (**B**) Plasma VDR levels of all patients and controls. Results are expressed as median with error bars (95% CI) and individual data points. Kruskal–Wallis H and Mann–Whitney U tests adjusted for multiple comparisons were used to compare values between groups. *p* < 0.05: statistically significant difference.

Flow cytometric analysis was performed with the addition of fluorescently labeled antibodies specific for the surface markers CD3 and CD14 of T lymphocytes and monocytes, respectively, to investigate the presence of different expression levels of VDR protein in specific cell subsets of PBMCs in patients and healthy controls. The levels of CD3+VDR+ and CD14+VDR+ cells were significantly increased in HCV+ patients and patients with liver cirrhosis compared with healthy controls (Figure 2A). Plasma VDR levels were significantly increased in cirrhotic patients compared with HCV+ patients and decreased in HCV+ patients compared with healthy controls (Figure 2B).

### 2.2. Expression of Genes Downstream of the Vitamin D-VDR Pathway in Healthy Controls Compared with HCV+ and Cirrhotic Patients

**Figure 3:**
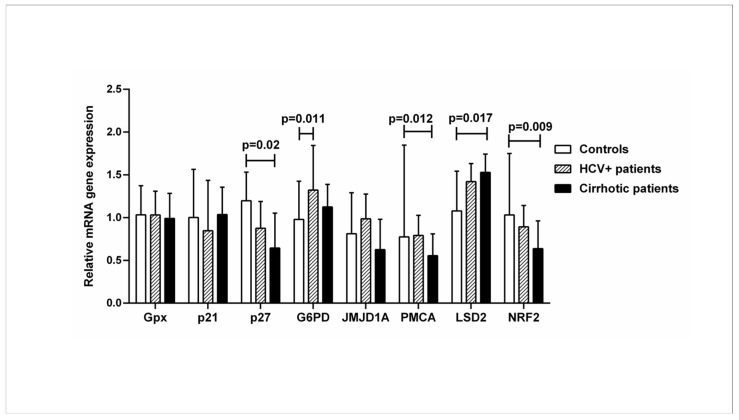
Relative mRNA expression of VDR-related genes downstream of the vitamin-VDR pathway in controls, HCV+, and cirrhotic patients. The mRNA levels were quantified by qPCR, and the values are expressed relative to the reference β-actin gene. Results are expressed as median with error bars (95% CI). Kruskal–Wallis H and Mann–Whitney U tests were used to compare values between groups and were adjusted for multiple comparisons. *p* < 0.05: statistically significant difference.

Gene expression analysis was performed in all subjects to quantify the mRNA of VDR-related genes downstream of the vitamin D-VDR pathway: *Gpx* (glutathione peroxidase 1), *p21* (cyclin-dependent kinase inhibitor 1), *p27* (cyclin-dependent kinase inhibitor 1B), *G6PD* (glucose-6-phosphate dehydrogenase), *JMJD1A* (lysine demethylase 3A), *PMCA* (plasma membrane calcium ATPase), *LSD2* (lysine demethylase 1B), and *NRF2* (nuclear factor-erythroid factor 2-related factor 2). The expression of *p27*, *PMCA*, and *NRF2* genes was significantly decreased in patients with liver cirrhosis compared to healthy controls, while the expression of the *LSD2* gene was increased. Expression of the *G6PD* gene was increased in HCV+ patients compared with healthy controls (*p* < 0.05) (Figure 3). 

The dysregulated expression of genes downstream of the vitamin D-VDR pathway involved in chromatin demethylation, reduction in oxidation, calcium regulation, cell cycle regulation, and modulation of multiple signaling pathways is observed in patients with chronic liver disease compared with healthy controls.

### 2.3. Relationship between VDR Expression Levels in Specific Cell Subsets and Liver Cirrhosis

Flow cytometric analysis was performed with the addition of fluorescently labeled antibodies specific for the surface markers CD3 and CD14 of T lymphocytes and monocytes, respectively, to determine the expression levels of VDR protein in specific cell subsets of PBMCs in patients and healthy controls. A moderate correlation was found between CD14+VDR+ cells and the MELD score (*p* < 0.05) (Table 3).

### 2.4. Relationship between VDR Polymorphisms and VDR Expression Levels in T Lymphocytes and Monocytes from Healthy Controls and HCV+ and Cirrhotic Patients

**Figure 4:**
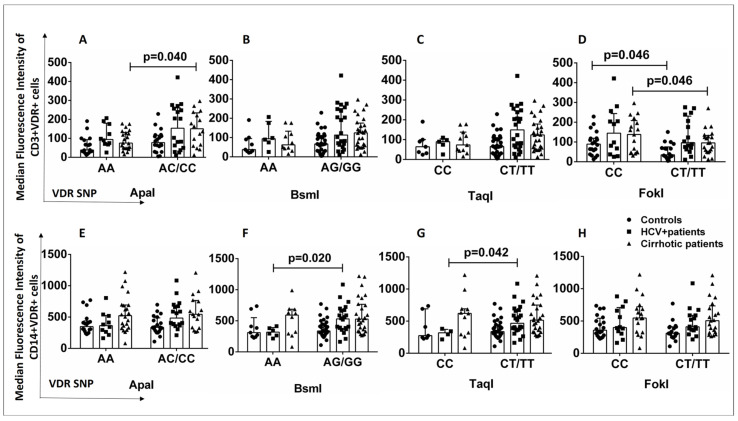
(**A**–**H**) Association of VDR SNPs with CD3+VDR+ and CD14+VDR+ cells in patients (cirrhotic and HCV+) and controls. Results are expressed as median with error bars (95% CI) and individual data points. Mann–Whitney U test was used to compare values between groups. *p* < 0.05: statistically significant difference.

Alterations in VDR protein levels (determined by flow cytometry) were also examined in patients and healthy controls in association with the presence of VDR polymorphisms because the presence of the latter could lead to altered VDR levels in specific cell subsets. The AA genotype of the ApaI SNP was associated with lower levels of CD3+VDR+ cells in the group of cirrhotic patients than in AC/CC carriers (Figure 4A). The AA (BsmI) and CC (TaqI) genotypes were associated with lower levels of CD14+VDR+ cells in HCV+ patients than in AG/GG and CT/TT carriers (Figure 4F,G). The CC genotype of the FokI SNP was associated with higher levels of CD3+VDR+ cells in cirrhotic patients and healthy controls than in CT/TT carriers (*p* < 0.05) (Figure 4D).

### 2.5. Relationship between VDR Polymorphisms and Plasma VDR Levels in Healthy Controls and HCV+ and Cirrhotic Patients

**Figure 5:**
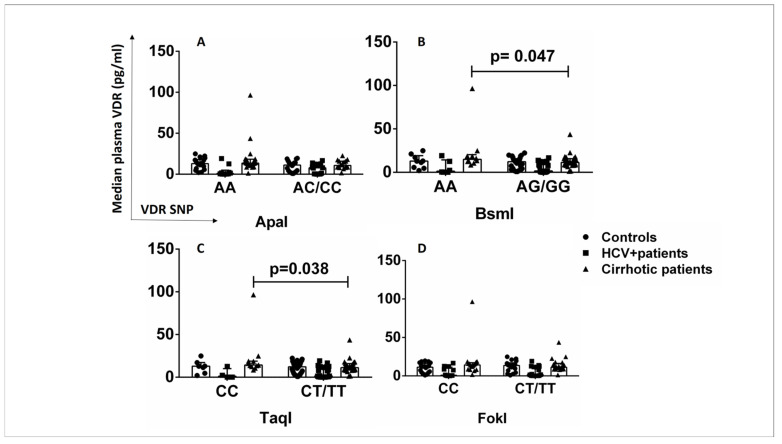
Association of VDR SNPs with VDR plasma levels in patients and controls. (**A**) VDR plasma levels in controls, HCV+ patients, and cirrhotic patients in association with ApaI SNP (AA vs. AC/CC). (**B**) VDR plasma levels in controls, HCV+ patients, and cirrhotic patients in association with the BsmI SNP (AA vs. AG/GG). (**C**) VDR plasma levels in controls, HCV+ patients, and cirrhotic patients in association with TaqI SNP (CC vs. CT/TT). (**D**) VDR plasma levels in controls, HCV+ patients, and cirrhotic patients in association with the FokI SNP (CC vs. CT/TT). Results are expressed as median with error bars (95% CI) and individual data points. Mann–Whitney U test was used to compare values between groups. *p* < 0.05: statistically significant difference.

The effects of VDR SNPs on VDR protein levels were also examined in plasma samples from patients and healthy controls to determine whether the association between VDR SNPs and VDR protein levels is cell-specific. The AA/CC genotypes of the SNPs BsmI and TaqI were associated with increased plasma VDR levels in cirrhotic patients compared with AG/GG and CT/TT carriers (*p* < 0.05) (Figure 5B,C). The SNPs ApaI and FokI were not associated with plasma VDR levels in any subgroup (Figure 5A,D). The presence of AA (BsmI) and CC (TaqI) genotypes in cirrhotic patients might have a different, non-cell-specific impact on VDR expression levels than in HCV+ patients, in whom the SNPs BsmI and TaqI showed a cell-specific association with lower CD14+VDR+ levels.

### 2.6. Relationship between VDR SNPs and Expression of Genes Downstream of the Vitamin D-VDR Pathway in Healthy Controls and HCV+ and Cirrhotic Patients

**Figure 6:**
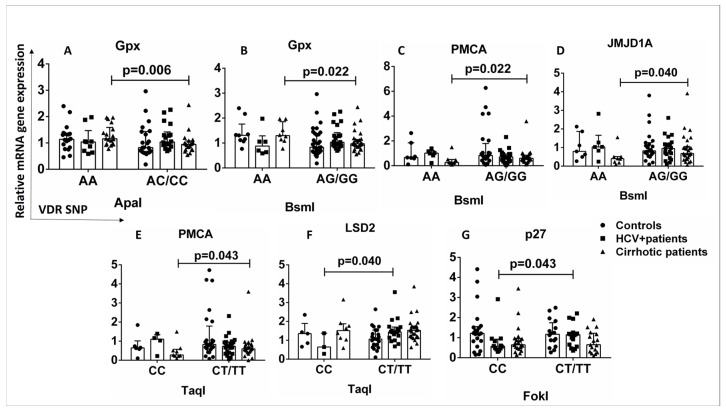
Association between VDR SNPs and expression of genes downstream of the vitamin D-VDR pathway. The mRNA levels were quantified by qPCR and the values are expressed relative to the reference gene β-actin. (**A**) Relative mRNA *Gpx* gene expression in association with ApaI SNP (AA vs. AC/CC) in controls and HCV+ and cirrhotic patients. (**B**) Relative mRNA *Gpx* gene expression in association with BsmI SNP (AA vs. AG/GG) in controls and HCV+ and cirrhotic patients. (**C**) Relative mRNA *PMCA* gene expression in association with BsmI SNP (AA vs. AG/GG) in controls and HCV+ and cirrhotic patients. (**D**) Relative mRNA *JMJD1A* gene expression in association with BsmI SNP (AA vs. AG/GG) in controls and HCV+ and cirrhotic patients. (**E**) Relative mRNA *PMCA* gene expression in association with TaqI SNP (CC vs. CT/TT) in controls and HCV+ and cirrhotic patients. (**F**) Relative mRNA *LSD2* gene expression in association with TaqI SNP (CC vs. CT/TT) in controls and HCV+ and cirrhotic patients. (**G**) Relative mRNA *p27* gene expression in association with FokI SNP (CC vs. CT/TT) in controls and HCV+ and cirrhotic patients. Results are expressed as median with error bars (95% CI) and individual data points. Mann–Whitney U test was used to compare values between groups. *p* < 0.05: statistically significant difference.

The binding of vitamin D to VDR leads to the induction or repression of a large number of target genes in various cell types, and the expressed proteins are involved in a variety of biological processes. The mRNA quantification of VDR-related genes downstream of the vitamin D-VDR pathway was performed to investigate the differences in mRNA levels of VDR-related genes in patients and healthy controls in the presence or absence of VDR SNPs. The AA genotype of ApaI and BsmI SNPs was associated with increased expression of the *Gpx* gene compared with AC/CC, AG/GG carriers in cirrhotic patients (Figure 6A,B). The AA genotype of the BsmI SNP was associated with decreased expression of the *PMCA* and *JMJD1A* genes compared to AG/GG carriers in cirrhotic patients (Figure 6C,D). The CC genotype of the TaqI SNP was associated with decreased expression of the *PMCA* gene compared with CT/TT carriers in cirrhotic patients (*p* < 0.05) (Figure 6E).

HCV+ patients with the CC genotype of TaqI SNP showed decreased expression of the *LSD2* gene compared to CT/TT carriers (Figure 6F), and the CC genotype of FokI SNP was associated with decreased expression of the *p27* gene compared to CT/TT HCV+ carriers (*p* < 0.05) (Figure 6G). In both HCV+ and cirrhotic patients, the presence of VDR SNPs is associated with the expression of genes regulating cell proliferation, cell differentiation, oxidative stress, metabolic processes, and intracellular signaling.

### 2.7. ApaI SNP Is Associated with Increased Liver Stiffness in HCV+ Patients

**Figure 7:**
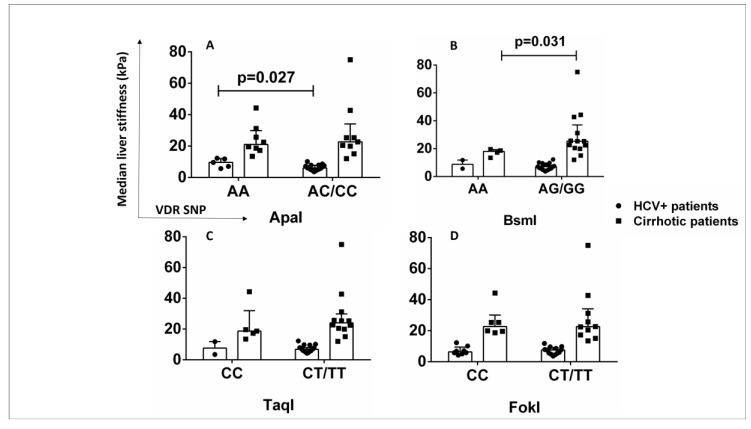
Association of VDR SNPs with liver stiffness in HCV+ and cirrhotic patients. (**A**) Median liver stiffness (kPa) associated with ApaI SNP (AA vs. AC/CC). (**B**) Median liver stiffness (kPa) associated with BsmI SNP (AA vs. AG/GG). (**C**) Median liver stiffness (kPa) associated with TaqI SNP (CC vs. CT/TT). (**D**) Median liver stiffness (kPa) associated with FokI SNP (CC vs. CT/TT). Results are expressed as median with error bars (95% CI) and individual data points. Mann–Whitney U test was used to compare values between groups. *p* < 0.05: statistically significant difference.

The influence of VDR SNPs on laboratory findings and clinical outcomes of cirrhotic and HCV+ patients was investigated. HCV+ patients with genotype AA of ApaI SNP had significantly increased liver stiffness (9.7 kPa (IQR 7.1–11.8)) compared to AC/CC carriers (6.05 kPa (IQR 5–7.7)) (*p* < 0.05) (Figure 7A). Cirrhotic patients with genotype AA of BsmI had significantly lower liver stiffness (18 kPa (IQR 15.4–19.15)) compared with AG/GG carriers (25.4 kPa (IQR 20.5–31.2)) (*p* < 0.05) (Figure 7B). No association was found between TaqI and FokI SNPs and liver stiffness in cirrhotic and HCV+ patients (Figure 7C,D). The ApaI SNP might have an impact on the progression of fibrosis in HCV+ patients.

### 2.8. Bioinformatics Analysis of the Interaction Affinity between VDR Protein and Vitamin D in the Absence (VDR wt Isoform) and Presence of the FokI VDR Polymorphism (VDR FokI Isoform)

#### 2.8.1. VDR Vitamin D-Binding Domain

**Figure 8:**
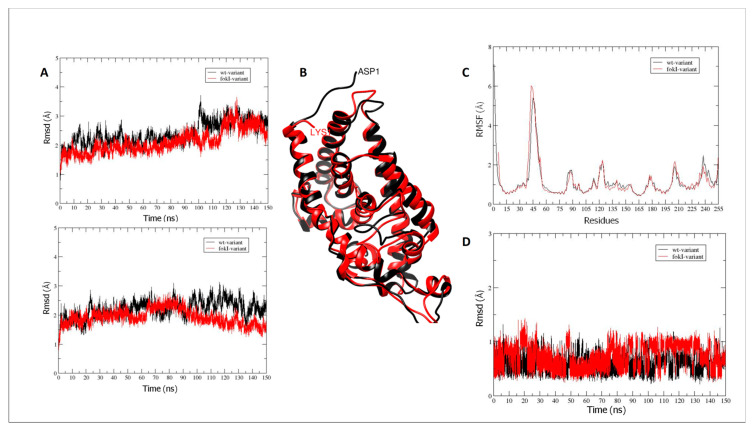
(**A**) Rmsd values for VDR vitamin D-binding domain backbone atoms (C, O, N, and Cα) during MD simulations for the wt (black) and fokI (red) variants in (**A**) (**top**) the apo form and (**bottom**) in complex with vitamin D. (**B**) Overlay of the representative clusters for the VDR vitamin D-binding domain for the wt (black) and fokI (red) isoforms. (**C**) Atomic fluctuations (root mean square fluctuations—rmsf) of protein residues in the apo form for wt (black) and the fokI (red) variant. (**D**) Rmsd values across the MD simulation for vitamin D in the binding cleft in complex with the wt (black) and the fokI (red) isoforms.

MD production simulation for the wt and fokI isoforms in the apo form shows the conformational changes in the vitamin D-binding domain of the receptor (Figure 8A (top), black and red, respectively). The Rmsd values for the backbone atoms (C, O, N, and Cα) do not show much difference in conformation compared with the initial structure of the VDR (mean Rmsd value of 2.35 Å). The difference between the wt and fokI isoforms is the absence of five residues at the N-terminus of the fokI variant (Figure 8B). Thus, the missing residues at the beginning of the polypeptide chain of the vitamin D-binding domain do not have a major effect on the conformation of the vitamin D-binding site and the overall movement of the domain. The lack of major conformational changes between the wt and fokI isoforms is also reflected in the clustering (Figure 8B) and atomic fluctuation (Figure 8C) analyses. In both cases, similar patterns (Figure 8C) are observed over the course of the MD simulations for the different regions of the polypeptide chain. Clustering analysis shows the presence of a distinct cluster for both isoforms (Figure 8B), a result consistent with the small changes in Rmsd values in Figure 8A. Similar patterns were observed in the analysis of the VDR in complex with vitamin D. Over the course of the MD simulations, the conformational changes of the polypeptide chain are similar for both the wt and fokI isoforms (Figure 8A (bottom), black and red, respectively). The vitamin D molecule in the binding cavity of the domain also does not show major conformational changes (Figure 8D). The behavior of the ligand can be attributed to the location of the binding cleft. Since it is located deep in the binding domain of VDR, the cavity does not allow much flexibility for the ligand.

#### 2.8.2. Interactions between Hydrogen Bonds (Hb)

**Figure 9:**
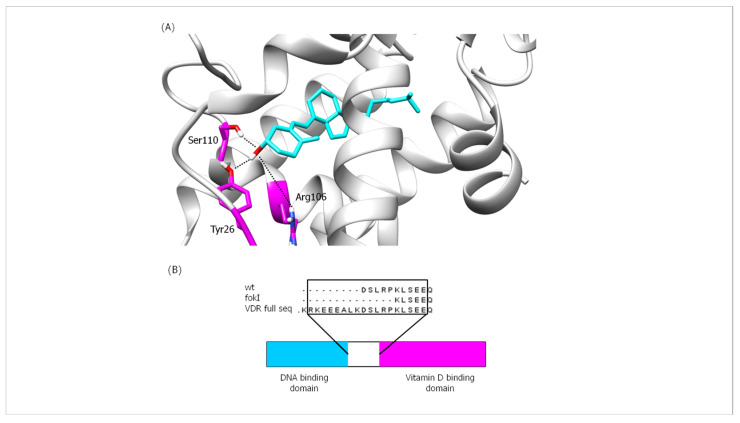
(**A**) Illustration of important Hb interactions between vitamin D and VDR in both isoforms (wt and fokI) and (**B**) schematic representation of VDR (**bottom**) and comparison of residual sequences of the linker region (**top**) for the different VDR variants.

Vitamin D binds to the ligand-binding domain (LBD) of VDR, which consists of 13 α-helices and a β-sheet forming a three-layer sandwich structure stabilized by 10 residues. Thirty-six amino acid residues are located in the ligand-binding pocket (LBP) of VDR, and six of these residues (Ser69, Arg106, Tyr26, Ser110, His137, and His229) are linked to the vitamin D ligand by hydrogen bonds [39]. The results of the analysis of the interactions in the vitamin D-VDR domain complexes (wt and fokI variants) are shown in the Supplementary Material (Table S1). In both isoform complexes, the most important Hb interaction is observed between residue Ser110 and vitamin D (Figure 9A and Table S1). This particular interaction is present during most of the simulation time. Another Hb that may play an important role in the binding of vitamin D to VDR involves Tyr26 in VDR. This particular Hb occurs mainly after the first ~60 ns of the simulation and persists for the rest of the production run. As shown in Figure 9A, both Ser110 and Tyr26 contribute to the anchoring of vitamin D in the binding cleft by interacting with the –OH moiety of vitamin D. The anchoring of vitamin D in the binding cleft is likely further enhanced by interactions with the side chain of Arg106. Analysis of the trajectories showed that the conformational changes of the vitamin D-binding domain of VDR between the wt and fokI variants are not extensive. As described above, there are no significant conformational changes for the vitamin D domain. This observation can be explained by the position of the changes resulting from the expression of the fokI polymorphism. As shown in Figure 9B, the fokI variant has a shorter linker connecting the DNA and vitamin D-binding domains. The shorter length of the linker region may affect the functionality of the DNA-binding domain by limiting its flexibility and movement. This suggests that the SNP is not directly involved in vitamin D binding; instead, it affects vitamin D reactivity through another regulatory mechanism involving the DNA-binding domain.

## 3. Discussion

In this study, we investigated the role of VDR in liver cirrhosis and HCV infection, focusing on the influence of the SNPs ApaI, BsmI, TaqI, and FokI in the VDR gene. A major objective of this study was to investigate the biological role of VDR SNPs in patients with two closely related liver diseases and to test the hypothesis that these genetic factors may play distinct biological roles in the progression of HCV infection to cirrhosis. We found that BsmI and TaqI SNPs had a differential effect on VDR levels in HCV infection and cirrhosis, as cirrhotic patients carrying BsmI and TaqI SNPs had significantly increased plasma VDR levels, and BsmI and TaqI SNPs were associated with significantly decreased VDR levels in CD14+ cells of HCV+ patients. The VDR SNPs also showed a greater impact on gene expression downstream of the vitamin D-VDR pathway in cirrhotic patients compared with HCV+ patients. Considering the lack of literature on the biological mechanisms of VDR SNPs in chronic liver disease and the need for further studies, we could hypothesize that VDR SNPs might play different biological and clinical roles in disease progression depending on the stage of chronic liver disease.

Plasma VDR levels were increased in cirrhotics carrying SNPs BsmI and TaqI, indicating a possible influence of SNPs BsmI and TaqI on VDR expression and release. SNPs in the VDR gene may have an impact on VDR transcription and expression. The SNPs ApaI and BsmI do not affect the amino acid sequence or structure of the VDR protein because they are located in intron 8 at the 3′ end of the VDR gene. However, the ApaI and BsmI SNPs could affect the alternative splicing of VDR mRNA, alter mRNA stability, and modulate the transcription of the VDR gene [40,41]. The TaqI polymorphism is located in exon 9 at the 3′ end of the human VDR gene and does not alter the VDR protein but causes a synonymous change due to a nucleotide substitution. The TaqI SNP may be involved in regulating the stability of VDR mRNA [42]. The FokI polymorphism has a direct effect on the VDR peptide chain because it is located in exon 2, the start codon of the VDR gene, resulting in an alternative translation start site and a VDR protein shortened by three amino acids [43].

The observed decrease in VDR expression levels in T lymphocytes from cirrhotic patients with the SNP ApaI indicates a possible negative impact of this SNP on the immunomodulatory activity of VDR by impairing VDR gene expression. The presence of the ApaI SNP has been associated with increased levels of pro-inflammatory cytokines (IL-1β and IL-8) in cirrhotic patients [38]. VDR deficiency has been shown to exacerbate inflammatory responses in the liver by promoting liver macrophage infiltration and increasing gene expression and systemic levels of pro-inflammatory cytokines, including IL-1β, IL-6, and TNF-α [44]. Vitamin D-VDR signaling has also been shown to downregulate the production of the pro-inflammatory cytokine IL-8 in monocytes [45]. The increased VDR expression in T lymphocytes from cirrhotic patients with the CC genotype of the FokI SNP may reflect a positive influence of FokI SNP on the immunomodulatory and antifibrotic activity of the vitamin D-VDR pathway in liver cirrhosis. The presence of FokI SNP has been associated with decreased levels of the pro-inflammatory cytokine IL-1β and has been reported as an independent prognostic factor for survival in patients with liver cirrhosis [38].

Vitamin D-VDR signaling has been shown to regulate inflammatory responses by enhancing the antibacterial activity of innate immune cells and controlling the proliferation and pro-inflammatory phenotypes of CD4+ T lymphocytes [46]. Moreover, VDR activity has been reported to inhibit liver fibrosis by suppressing the expression of pro-fibrotic genes mediated by TGF-β1/SMAD signaling [47]. Therefore, cirrhotic patients with the FokI SNP who express the VDR protein at higher levels may have lower pro-inflammatory and pro-fibrotic responses and thus better disease course and lower progression to fibrosis due to increased VDR activity compared to patients who do not carry the CC (FokI) genotype. However, the association between the FokI SNP and the number of CD3+VDR+ T cells in peripheral blood was also observed in healthy controls, suggesting that the effect of the FokI SNP on VDR is not unique to cirrhotics.

Regarding the decrease in VDR expression in monocytes in the presence of BsmI and TaqI SNPs in HCV+ patients in our study, there are few data showing a correlation between VDR SNPs and VDR protein levels. The BsmI and TaqI SNPs have been strongly associated with the degree of fibrosis in chronic hepatitis C patients and have been proposed as markers for disease evaluation [48]. The TaqI SNP has been associated with decreased expression of the VDR gene, which has a strong fibrogenic effect on the human intestine, as it has been linked to increased fibroblast proliferation and increased expression of the collagen 1A1 gene [49]. The SNPs BsmI and TaqI could potentially contribute to a worsening of the clinical course of hepatitis C by downregulating VDR expression and thus its immunomodulatory activity. The vitamin D-VDR system is thought to play an important role in the progression of metabolic and viral chronic liver injury. In patients with chronic hepatitis C, portal vein inflammation is significantly higher in patients with VDR-negative inflammatory cells and low VDR expression in hepatocytes [50]. Low VDR protein expression has also been associated with severe necroinflammatory activity and severe fibrosis in patients with genotype 1 chronic hepatitis C [51].

The potent effect of VDR SNPs on VDR expression levels may play a role in liver disease progression, considering the observed inverse relationship between CD14+VDR+ cell levels and MELD score in the cirrhotic patients in our study. The anti-inflammatory and antifibrotic effects of VDR on liver tissue could probably explain this negative correlation [5]. A correlation between the systemic inflammatory syndrome and a high MELD score was also found in hospitalized cirrhotic patients, suggesting a relationship between inflammatory responses and the severity of liver disease [52]. 

VDR is a nuclear transcription factor that binds to the vitamin D response element (VDRE) and triggers the transcription of downstream target genes that mediate the biological activity of the receptor. We analyzed the gene expression of VDR-related genes downstream of the vitamin D-VDR pathway in all study participants to investigate the possible effects of VDR SNPs on gene expression. The presence of ApaI, BsmI, and TaqI SNPs in cirrhotic patients may have an impact on the progression of fibrosis and liver necrosis in cirrhosis, as it is associated with the expression of *Gpx*, plasma membrane Ca^2+^ ATPase (*PMCA*), and Jumonji domain containing 1A (*JMJD1A*) genes, which are involved in biological processes contributing to hepatocyte function, chronic liver injury, and fibrosis. The enzyme Gpx has peroxidase activity that protects the organism from oxidative damage. The excessive production of reactive oxygen species (ROS) leads to oxidative stress, which can disrupt hepatic homeostasis and has been associated with liver disease and other chronic and degenerative disorders [53,54]. Oxidative stress contributes to liver injury by causing irreversible changes in lipid, protein, and DNA content and modulating metabolic pathways involved in normal biological functions. Complicated interactions between pathological factors, inflammation, ROS, and immune responses have also been suggested [54,55]. Lipid peroxidation triggered by oxidative stress induces hepatic stellate cell proliferation and collagen synthesis [56,57]. PMCA is an important transport protein in the plasma membrane of cells that regulates intracellular Ca^2+^ concentration. Calcium-mediated processes regulate many functions in liver tissue such as cell growth, glucose production, and bile secretion, and Ca^2+^ also plays a role in apoptotic and necrotic death. Given the importance of Ca^2+^ in liver physiology, the regulation of intracellular Ca^2+^ concentration is critical for normal hepatocyte function and survival [58]. Tight regulation of Ca^2+^ levels appears to be important for balanced immune responses related to the mobilization of naïve and memory T cells [59]. JMJD1A (Jumonji domain containing 1A) is a histone demethylase that has been shown in vitro and in vivo to modulate the activation of HSCs and liver fibrosis by affecting peroxisome proliferator-activated receptor gamma (*PPARγ*) gene expression. The suppression of *JMJD1A* gene expression has been shown to significantly increase α-smooth muscle actin and *Col1a* expression, collagen production, and necrosis [60] (Figure 1). 

The presence of TaqI and FokI SNPs in HCV+ patients may have an impact on liver cell metabolic plasticity and liver cancer development, considering that they are linked to the expression of genes encoding lysine-specific histone demethylase 2 (LSD2) and cyclin-dependent kinase inhibitor 1B (p27). LSD2 catalyzes histone demethylation, thereby affecting gene expression and chromatin function [61]. LSD2 has been shown to maintain energy balance in liver cells by suppressing lipid flux and metabolism [62]. Aberrant LSD2 gene expression may be associated with its dysregulated histone activity, which contributes to aberrant gene expression in cancer [63]. p27 is an enzyme inhibitor that controls cell cycle progression. The regulation of *p27* gene expression is thought to be involved in controlling the cell cycle progression of quiescent mature hepatocytes during hepatocyte differentiation and proliferation [64]. p27 has been characterized as a tumor suppressor by blocking cell cycle progression and preventing rapid and abnormal cell division [65,66]. In HCC patients, decreased p27 gene expression has been associated with increased tumor size and cell proliferation [67] (Figure 1). 

The molecular mechanism by which VDR SNPs might affect VDR expression and cell activity remains to be elucidated. Using molecular dynamics simulations, we were able to investigate a possible effect of the FokI SNP on the binding of vitamin D to VDR. The FokI SNP is the only reported VDR polymorphism that has a direct effect on the VDR peptide chain compared with ApaI, BsmI, and TaqI. A comparison of the major Hb interactions between vitamin D and the VDR protein in both isoforms (wt and fokI) by MD simulation revealed no significant conformational changes in the vitamin D-binding domain of the VDR protein between the wt and fokI isoforms. This observation could be explained by the position of the changes attributable to the FokI SNP in the VDR protein. The presence of the FokI SNP results in the deletion of amino acid residues located at the beginning of the polypeptide chain of the vitamin D-binding domain and thus does not significantly affect the conformation of the vitamin D-binding site and the overall movement of the domain. In addition, the vitamin D molecule is located deep within the VDR-binding domain, so the cavity does not provide much flexibility for the ligand to interact with other parts of the peptide chain. The SNPs ApaI, BsmI, and TaqI have no effect on the VDR polypeptide chain. Most common polymorphisms in noncoding regions have been characterized as regulatory polymorphisms that can affect transcription [68], RNA splicing, stability, or translation [69]. Although the FokI SNP has a direct effect on VDR protein, it results in changes that may not affect vitamin D binding to VDR. The fokI variant has a shorter linker connecting the DNA and vitamin D-binding domains, which may affect the functionality of the DNA-binding domain by limiting its flexibility and movement. The functional impact of individual SNPs may be minimal, but a haplotype consisting of a set of polymorphisms that are in linkage disequilibrium could be associated with a functional outcome related to gene expression or function [70]. Future genome-wide studies in combination with in vitro studies focusing on the effects of the synergistic action of VDR SNPs on biological functions in liver cells will provide useful information on the functional dynamics of specific VDR SNPs, involving a wide range of samples from the general population.

Finally, we examined the effects of VDR SNPs on the laboratory and clinical outcomes of the patients in our study. The AA genotype of the ApaI SNP was associated with increased liver stiffness in HCV+ patients, indicating a possible negative impact of the ApaI SNP on liver fibrosis. The ApaI SNP, as a member of the grouped SNPs (bAt (CCA) haplotype consisting of the BsmI, ApaI, and TaqI SNPs), has been associated with increased fibrosis progression in chronic hepatitis C [35,36] and inflammation in cirrhosis. Cirrhotic patients carrying the AA genotype had higher levels of pro-inflammatory cytokines (IL-1β, IL-8) compared with AC/CC carriers [38]. The presence of the BsmI SNP was associated with decreased liver stiffness in cirrhotic patients, an observation that may be related to the increased plasma VDR levels in cirrhotic patients carrying the BsmI SNP. The elevated plasma VDR levels may indicate increased VDR activity, which has been reported to inhibit liver fibrosis by suppressing the TGF-β1/SMAD signaling-mediated expression of pro-fibrotic genes [47]. We also observed significant differences in plasma VDR levels between patients and controls. Plasma VDR was increased in cirrhotic patients compared with controls and HCV+ patients and decreased in HCV+ patients compared with controls. There is a dynamic spectrum of immunological disorders that develop during liver cirrhosis [71]. Chronic HCV infection is associated with T cell exhaustion and CD4+ T cell deletion, which may lead to immune escape of the virus [72]. We could hypothesize that the increased plasma VDR levels in cirrhotic patients in our study compared with the plasma VDR levels of controls and HCV+ patients are due to the increased inflammatory responses that activate the VDR receptor in T lymphocytes to modulate inflammation [73]. In contrast, severe T cell exhaustion in HCV patients might be associated with a decrease in T cell activation and, consequently, VDR protein levels. However, we must keep in mind that plasma VDR also represents VDR protein from other tissues, and other factors may be involved in this outcome.

Our study has certain limitations, including the relatively small sample size in all groups, the lack of grouping of patients by different stages of liver disease, and the lack of measurements of vitamin D levels. Although we have demonstrated to some extent the influence of VDR SNPs on VDR protein levels and transcription of VDR-related genes in patients with chronic liver disease, future studies will elucidate the precise molecular mechanisms that determine the biological effects of VDR SNPs on liver tissue and pathophysiology.

## 4. Materials and Methods

### 4.1. Patients

A total of 96 patients with chronic liver disease and 43 healthy controls were recruited for this study between October 2018 and December 2020. Peripheral blood samples were collected from all participants during this period. A total of 51 patients had cirrhosis of various etiologies (alcohol consumption 31.4%, HCV infection 15.7%, alcohol + HCV 11.8%, HBV ± HDV infection 9.8%, nonalcoholic steatohepatitis 9.8%, alcohol + HBV 9.8%, autoimmune hepatitis 3.9%, other 5.9%, and primary biliary cirrhosis 2%), and 45 patients had HCV infection. Patients were followed at the Hepatology Clinic of Patras University Hospital (PUH) at regular intervals according to standard clinical practice for monitoring patients with liver disease. 

All study participants gave written informed consent before participating in the study. The study protocol was approved by the Scientific Review Board (Re: 1058/28-11-18) and the Ethics Committee (Re: 773/13-11-18) of PUH. PUH adheres to the Declaration of Helsinki on Ethical Principles for Medical Research Involving Human Subjects. 

All patients and healthy controls were questioned about their intake of vitamin D supplements, and it was confirmed that they did not take vitamin D supplements during the recruitment and follow-up periods.

**Figure 10:**
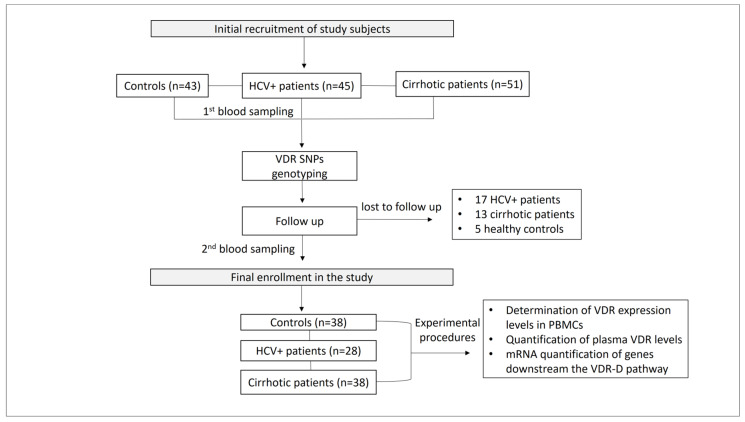
Study workflow.

Patient monitoring included a complete medical history, etiology of liver disease, CP score, MELD score for the severity of chronic liver disease, and the diagnostic method used. The exclusion criteria of the study were (1) age < 18 years, (2) patients with autoimmune and inflammatory diseases, obesity, and liver diseases other than cirrhosis and HCV, (3) refusal to sign the informed consent form, (4) participants lost to follow-up, and (5) death (Figure 10).

### 4.2. DNA Extraction

Genomic DNA was extracted using the NucleoSpin Blood QuickPure kit (Macherey-Nagel, Düren, Germany). The DNA concentration of the samples was determined using a Nanodrop spectrophotometer (UV Spectrophotometer Q3000, Quawell Technology, Inc., San Jose, CA, USA).

### 4.3. Genotyping of VDR Polymorphisms

Genotyping of VDR polymorphisms was performed using TaqMan SNP genotyping assays (Applied Biosystems; Foster City, CA, USA). PCR reactions were performed in MicroAmp Fast Optical 96-well Reaction Plates (Applied Biosystems) on the Step One Plus real-time PCR system (Applied Biosystems, CA, USA). Predesigned TaqMan SNP genotyping assays (Applied Biosystems) were used to design the probes rs731236 (TaqI), rs1544410 (BsmI), rs7975232 (ApaI), and rs2228570 (FokI). Each plate contained two wells with healthy non-template controls. PCR cycle parameters for DNA amplification were as follows: 95 °C for 10 min, followed by 40 cycles of 95 °C for 15 s and 60 °C for 1 min.

### 4.4. Flow Cytometry Analysis of Peripheral Blood Mononuclear Cells

Peripheral blood mononuclear cells (PBMCs) were analyzed by flow cytometry to determine the relative concentrations of CD3+VDR+ and CD14+VDR+ cells, using a BD FACS CaliburTM flow cytometer. PBMCs were isolated from whole blood (8–10 mL) by centrifugation over a Ficoll-Paque gradient (Biowest SAS, Nuaillé, France). PBMCs (10^6^ cells/experimental point) were incubated with anti-CD3 and anti-CD14 monoclonal antibodies PE-CyTM5 Mouse Anti-Human CD3, clone UCHT1, RUO (BD Biosciences, Franklin Lakes, NJ, USA), and FITC Mouse Anti-Human CD14, clone M5E2, RUO (BD Pharmingen). Cells were then fixed and permeabilized (BD Biosciences Cytofix/Cytoperm Fixation and Permeabilization Solution, RUO, BD Perm/Wash Buffer, RUO) and stained with a mouse anti-human VDR monoclonal antibody (clone D-6, Santa Cruz Biotechnology, Inc., Dallas, TX, USA). Analysis of the results was performed using FlowJo™ v10 software. A detailed description of the flow cytometric analysis can be found in the Supplementary Material (Figures S1–S3).

### 4.5. Enzyme-Linked Immunosorbent Assay (ELISA)

Plasma was obtained by centrifugation of peripheral blood at 2000× *g* for 10 min (15–24 °C) within 30 min of blood collection. Plasma VDR concentration (ng/mL) (pVDR) was quantified by ELISA using the Human Vitamin D Receptor (VDR) ELISA kit CSB-E05136h, Cusabio, USA. The pVDR concentration was measured using the microplate reader Varioskan™ LUX (Thermo Fisher Scientific, Waltham, MA, USA).

### 4.6. Quantitative Real-Time PCR (qPCR)

Total RNA was extracted from PBMCs using a TRIzol reagent (Sigma-Aldrich, St. Louis, MO, USA). Total RNA was subsequently reverse transcribed into cDNA using the M-MLV Reverse Transcriptase kit (Sigma-Aldrich). Relative mRNA levels of downstream genes of the VDR-D pathway were quantified by qPCR using the KAPA SYBR FAST qPCR Master Mix (2X) Universal kit (Kapa Biosystems, Inc., Cape Town, South Africa) in the Step One Plus qPCR system (Applied Biosystems, Foster City, CA, USA). Data were analyzed using the Step One Plus qPCR system software (Applied Biosystems). *β-Actin* served as an internal reference gene. All measurements were performed in triplicate. The values obtained were normalized to the endogenous *β-actin* gene of the healthy controls and calculated using a comparative Ct method for relative quantification (2^−ΔΔCt^). PCR cycle parameters and primers for each gene are listed in Table 4. 

### 4.7. Structure Preparation and Molecular Dynamics (MD) Simulations

An analysis of the interactions in the vitamin D-VDR domain complex in the absence and presence of the FokI variant was performed to evaluate the structural effects of the FokI variant on the binding of vitamin D to the VDR using MD simulations.

Structure preparation: The crystallographic structure with PDB code 1ie91 was used to generate the parameters for the wild-type (wt) domain of the VDR and the FokI variant. USCF Chimera (v1.11.2) [74] was used to design the vitamin D molecule optimized for ab initio calculations using GAMESS software version 2019 R1 [75].
  MD simulations: MD simulations were performed using AMBER14 software [76]. The ff14SB [77] and GAFF [78] force fields were used to assign partial atomic charges for the amino acid residues and the vitamin D molecule, respectively. A detailed description of the methods used for the design and MD simulations [79,80,81,82,83,84,85,86,87,88,89,90,91,92,93] can be found in the Supplementary Material (Section S4).

### 4.8. Statistical Analysis

Continuous variables were presented as medians and interquartile ranges (IQRs), whereas categorical variables were presented as numerical values (n) and corresponding percentages. Data sets for CD3+, CD14+, CD3VDR+, CD14VDR+, pVDR values and expression levels of VDR-related genes were not normally distributed according to the Shapiro–Wilk normality test. Therefore, all comparisons were performed with nonparametric tests. Pearson’s chi-square test or Fisher’s exact test were used for comparison between categorical data when applicable. Pearson’s chi-square test with one degree of freedom was performed to assess the deviation from Hardy–Weinberg equilibrium (HWE) for each SNP in the total population and subgroups. The Mann–Whitney U test was used to compare medians between two groups. The Kruskal–Wallis H test was used for comparisons between three groups. Following a significant Kruskal–Wallis test, a pairwise Mann–Whitney U test was performed to detect specific group differences, with the *p* value adjusted for false positives by multiplying by the number of pairs tested (Bonferroni method). Spearman’s correlation coefficient was used to estimate the degree of association between two variables. Statistical analysis was performed using the statistical software package IBM SPSS version 26.0. Two-tailed *p* values <0.05 were considered statistically significant.

## 5. Conclusions

The clinical severity of liver disease is associated with VDR levels. Therefore, SNPs in the VDR gene may be associated with the progression of chronic liver disease by affecting VDR protein levels. The effect of SNPs on the expression of VDR-related genes downstream of the vitamin D-VDR pathway, which are involved in important biological processes for normal hepatocyte function and immune homeostasis, is another test point for the potential effects of VDR SNPs on chronic liver disease progression. While the relationship between VDR SNPs and VDR function and its impact on the pathophysiology of chronic liver disease is not well understood, our study sheds light on how nucleotide changes might influence the immune response and potentially contribute to the progression of fibrosis via alterations in VDR expression levels, altered binding between VDR and vitamin D, and altered VDR-related gene expression. These findings could have important implications for the clinical monitoring of high-risk patients and for pharmacogenetic research to develop personalized treatments. By considering the role of VDR-SNPs, we may be able to better treat patients with chronic liver disease according to their genetic profile and improve disease outcomes.

## Figures and Tables

**Table 1:** Demographic and baseline characteristics of patients and healthy controls.

	Healthy Controls		Cirrhotic Patients		HCV+ Patients	
	*n*= 38		*n* = 38		*n* = 28	
	*n*	%	*n*	%	*n*	%
Sex (M/F)	22/16	57.9/42.1	26/12	68.4/31.6	16/12	57.1/42.9
Genotype (1/3/4)	-	-	-	-	8/11/7	30.7/42.3/26.9
Child Pugh stage (A/B/C)	-	-	29/8/1	76.3/21.1/2.6	-	-
Decompensation development (yes/no)	-	-	9/29	23.7/76.3	-	-
HCC development (yes/no)	-	-	6/32	15.8/84.2	-	-
	Median	IQR	Median	IQR	Median	IQR
Age (years)	53.5	48–64.25	56.5	51–66	43.5	38–56.75
Hb (g/dL)	-	-	13.95	12.35–14.62	14.7	12.85–15.30
Plt (K/μL)	-	-	158,000	110,000–238,250	203,000	169,000–250,000
INR	-	-	1.09	0.96–1.23	0.94	0.90–1.04
Creatinine (mg/dL)	-	-	0.8	0.7–0.9	0.8	0.79–1
SGOT (U/L)	-	-	36	25–80.75	33.50	24.75–58.75
SGPT (U/L)	-	-	40	25–92.50	41	21.75–96.25
G-GT (U/L)	-	-	66	40.25–130.52	13.10	12.35–13.7
ALP (U/L)	-	-	107	82–141	65	52–83.50
Albumin (g/dL)	-	-	4.1	3.8–4.3	4.50	4.20–4.60
Total Bilirubin (mg/dL)	-	-	0.95	0.68–1.46	0.47	0.39–0.64
Liver Stiffness (kPa)	-	-	22.5	18–28.45	6.95	5.32–8.85
HCV RNA (IU/L)	-	-	-	-	122,500	28,500–528,000
Child–Pugh score	-	-	5.50	5–6.25	-	-
MELD score	-	-	7	6–9	-	-

HCC, Hepatocellular carcinoma; Hb, Hemoglobin; Plt, platelet; INR, international normalized ratio; SGOT, serum glutamic-oxaloacetic transaminase; SGPT, Serum glutamic pyruvic transaminase; G-GT, gamma-glutamyl transferase; ALP, Alkaline phosphatase; HCV, Hepatitis C virus; MELD, Model for End-stage Liver Disease.

**Table 2:** Distribution of VDR SNPs in patients (*n* = 66) and healthy controls (*n* = 38).

	**ApaI** (rs7975232)				**BsmI** (rs1544410)			
	SNP location	Nucleotide change	SNP alleles		SNP location	Nucleotide change	SNP alleles	
	Intron 8	A > C	A > a		Intron 8	A > G	B > b	
n (%)	**AA (AA)**	**AC (Aa)**	**CC (aa)**		**AA (BB)**	**AG (Bb)**	**GG (bb)**	
Controls	18 (47.4)	15 (39.5)	5 (13.2)	Reference group	9 (23.7)	18 (47.4)	11 (38.9)	Reference group
Cirrhotics	18 (47.4)	11 (28.9)	9 (23.7)	*p* = 0.448	9 (23.7)	14 (36.8)	15 (39.5)	*p* = 0.617
HCV+	9 (32.1)	15 (53.6)	4 (14.3)	*p* = 0.487	7 (25)	10 (35.7)	11 (39.3)	*p* = 0.628
	**TaqI** (rs731236)				**FokI** (rs2228570)			
	SNP location	Nucleotide change	SNP alleles		SNP location	Nucleotide change	SNP alleles	
	Exon 9	C > T	T > t		Eχon 2	C > T	F > f	
n (%)	**CC (TT)**	**CT (Tt)**	**TT (tt)**		**CC (FF)**	**CT (Ff)**	**TT (ff)**	
Controls	7 (18.4)	18 (47.4)	13 (34.2)	Reference group	21 (55.3)	15 (39.5)	2 (5.3)	Reference group
Cirrhotics	9 (23.7)	16 (42.1)	13 (34.2)	*p* = 0.875	21 (55.3)	13 (34.2)	4 (10.5)	*p* = 0.736
HCV+	7 (25)	10 (35.7)	11 (39.3)	*p* = 0.653	13 (46.4)	14 (50)	1 (3.6)	*p* = 0.702

SNP, single nucleotide polymorphism. Fisher’s exact test using 2 × 3 contingency tables was conducted to investigate the distribution of genotypes among the participants. *p* > 0.05 indicates no significant difference in the distribution of genotypes among the subgroups.

**Table 3:** Association between CD3+VDR+, CD14+VDR+ and plasma VDR levels and CP and MELD score in cirrhotic patients.

	CD3+VDR+ (MFI)		CD14+VDR+ (MFI)		Plasma VDR (pg/mL)	
	Spearman’s Rho	*p* Value	Spearman’s Rho	*p* Value	Spearman’s Rho	*p* Value
Child–Pugh (CP) score	−0.048	0.776	−0.100	0.549	0.188	0.259
MELD score	−0.197	0.236	−0.347	0.033	−0.010	0.955

MFI, median fluorescent intensity; MELD, Model for End-stage Liver Disease.

**Table 4:** PCR primers and thermal cycling parameters.

Gene	PCR primer (5′→3′)	PCR Product (bp)	PCR Cycling Parameters
*Gpx*	TATCGAGAATGTGGCGTCCC TCTTGGCGTTCTCCTGATGC	143	95 °C 3 min, 95 °C 3 s, 60 °C 30 s
*p21*	AGTCAGTTCCTTGTGGAGCC CATTAGCGCATCACAGTCGC	184	95 °C 3 min, 95 °C 3 s, 63 °C 30 s
*p27*	ACGTGCGAGTGTCTAACGG CGCCTCTTCCATGTCTCTGC	158	95 °C 3 min, 95 °C 3 s, 60 °C 30 s
*G6PD*	GAAACGGTCGTACACTTCGG CCGACTGATGGAAGGCATCG	153	95 °C 3 min, 95 °C 3 s, 60 °C 30 s
*JMJD1A*	CAGTTGCCTAAATGCCGA TGAATTGTAACCTCCTGAAGTG	112	95 °C 3 min, 95 °C 3 s, 61 °C 30 s
*PMCA*	CAGTTATGTGGGGACGAAATG GCGGTGAGTCTTGAGTAATGC	123	95 °C 3 min, 95 °C 3 s, 61 °C 30 s
*LSD2*	GCGTGCTGATGTCTGTGATT TTGTGGGATCTGGGACCTC	132	95 °C 3 min, 95 °C 3 s, 60 °C 30 s
*NRF2*	GCGCAGACATTCCCGTTTG GACTGGGCTCTCGATGTGAC	96	95 °C 3 min, 95 °C 3 s, 61 °C 30 s
*TRPV5*	TGGCACTGTTCACCACCTTT CAATGATGGCGAAGGCGAAG	114	95 °C 3 min, 95 °C 3 s, 62 °C 30 s

*Gpx*, glutathione Peroxidase 1; *p21*, cyclin-dependent kinase inhibitor 1; *p27*, cyclin-dependent kinase inhibitor 1B; *G6PD*, glucose-6-phosphate dehydrogenase; *JMJD1A*, lysine dimethylase 3A; *PMCA*, plasma membrane calcium ATPase; *LSD2*, lysine demethylase 1B; *NRF2*, nuclear factor-erythroid factor 2-related factor 2; *TRPV5*, transient receptor potential cation channel subfamily V member 5.

## Data Availability

The data presented in this study are available on request from the corresponding author. The data are not publicly available as they involve human subjects, and their confidentiality and ethical considerations must be respected.

## Supplementary Materials

The supporting information can be downloaded at: https://www.mdpi.com/article/10.3390/ijms241411404/s1.

## Abbreviations

VDR, vitamin D receptor; SNPs, single nucleotide polymorphisms; HCV, hepatitis C virus; MELD, model for end-stage liver disease; CP, Child–Pugh; PBMCs, peripheral blood mononuclear cells; MFI, median fluorescence intensity; pVDR, plasma VDR; MD, molecular dynamics; Gpx, glutathione Peroxidase 1; p21, cyclin-dependent kinase inhibitor 1; p27, cyclin-dependent kinase inhibitor 1B; G6PD, glucose-6-phosphate dehydrogenase; JMJD1A, lysine dimethylase 3A; PMCA, plasma membrane calcium ATPase; LSD2, lysine demethylase 1B; NRF2, nuclear factor-erythroid factor 2-related factor 2; TRPV5, transient receptor potential cation channel subfamily V member 5; RMSD, root mean square deviation.
