# Supplementary material for: The Influence of Single Nucleotide Polymorphisms on Vitamin D Receptor Protein Levels and Function in Chronic Liver Disease
Source: Int J Mol Sci. 2023 Jul 13;24(14):11404. doi: 10.3390/ijms241411404 (PMC10380285; doi:10.3390/ijms241411404)
Supplement: Supplementary file 1 [file ijms-24-11404-s001.zip › ijms-2440020-supplementary.pdf]

Supplementary Material

The Influence of Single Nucleotide Polymorphisms on Vitamin D Receptor Protein Levels and Function in Chronic Liver Disease

Evanthia Tourkochristou [1,2], Efthymios P. Tsounis [1], Haralampos Tzoupis [3], Ioanna Aggeletopoulou [1,2], Aggeliki Tsintoni [1], Theoni Lourida [1], Georgia Diamantopoulou [1], Konstantinos Zisimopoulos [1], Theodora Kafentzi [1], Anne-Lise de Lastic [2], Maria Rodi [2], Theodore Tselios [3], Konstantinos Thomopoulos [1], Athanasia Mouzaki [2,†] and Christos Triantos [1,*,†]

[1] Division of Gastroenterology, Department of Internal Medicine, Medical School, University of Patras, University Hospital of Patras, 26504 Patras, Greece; iaggel@hotmail.com (I.A.); agtsintoni@gmail.com (A.T.)

[2] Division of Hematology, Department of Internal Medicine, Medical School, University of Patras, 26504 Patras, Greece

[3] Department of Chemistry, University of Patras, 26504 Patras, Greece

* Correspondence: chtriantos@hotmail.com or chtriantos@upatras.gr

† These authors contributed equally to this work and share senior authorship.

Section S1. Vitamin D-VDR signaling pathways and regulation of downstream gene expression. Vitamin D exerts its biological effects by interacting with the VDR receptor through two distinct pathways: the genomic pathway and the nongenomic pathway. These pathways play a critical role in regulating downstream gene expression.

In the genomic pathway, vitamin D enters the cell by crossing the plasma membrane and subsequently binds to the VDR receptor in the cytoplasm. This binding event triggers activation of the VDR receptor. After activation, the VDR receptor forms a heterodimer with the retinoic acid receptor RXR. The next step is the translocation of the vitamin D-VDR-RXR complex from the cytoplasm to the nucleus. In the nucleus, this complex recognizes and binds to specific DNA sequences, called cis-DNA vitamin D response elements (VDREs), located in the gene promoters of the target genes. Binding of the vitamin D-VDR-RXR complex to VDREs serves as a regulatory signal that initiates transcription of downstream genes (1).

Binding of the heterodimer VDR-RXR to DNA has the potential to affect histone modifications, thereby affecting the accessibility of transcription factors to chromatin (2). High-affinity binding of vitamin D to the ligand-binding domain (LBD) region of VDR results in a conformational change in helix 12 of the C-terminus of VDR. This conformational change allows VDR to interact with various cofactors (3,4). Cyclin-dependent kinase inhibitor 1 (p21), which has a critical function in controlling cell cycle progression and growth, contains VDREs that allow it to be directly targeted by the D-VDR complex via the genomic pathway (5). Activation of gene expression of enzymes involved in DNA demethylation is an important mechanism contributing to the regulation of transcription and chromatin function. Two enzymes affected by the genomic vitamin D-VDR pathway are Jumonji domain containing 1A (JMJD1A) and lysine-specific demethylase 2 (LSD2). These enzymes play a critical role in catalyzing DNA demethylation, which involves the removal of methyl groups from DNA

molecules. By actively demethylating DNA, JMJD1A and LSD2 contribute to the maintenance of appropriate gene expression patterns and modulation of chromatin structure (6,7).

The nongenomic signaling pathway of vitamin D involves the activation of various signaling molecules and the rapid production of secondary messengers. Key players in this signaling pathway include phosphatidylinositol kinase-3 (PI3K), Akt kinase (also known as protein kinase B or PKB), and phospholipase C (PLC). In addition, the rapid production of secondary messengers such as cyclic AMP (cAMP) and calcium ions (Ca^{2+}) contributes to the signaling cascade. Once activated, these signaling molecules set in motion a series of events involving the activation of protein kinases. Examples of such protein kinases include mitogen-activated protein kinase (MAPK), protein kinase A (PKA), protein kinase C (PKC), proto-oncogenic tyrosine protein kinase (SRC), and Ca^{2+}-calmodulin kinase II (CAMKII). Nongenomic effects of vitamin D include the opening of Ca^{2+} channels leading to an increase in intracellular calcium levels and triggering downstream effects on cellular processes (8–10).

Protein kinases target transcription factors such as transcription factor specificity protein 1 (Sp1), Sp3, and RXR and activate transcription by binding to VDRE elements in gene promoters. Vitamin D also exerts nongenomic effects by regulating the binding of VDR to proteins such as STAT1 and IKKβ, which allows cross-regulation of gene expression (11). The vitamin D-VDR pathway exerts indirect control over gene expression by affecting other signaling molecules, pathways, and transcription factors. One example is the suppression of the glutathione peroxidase (Gpx) gene through vitamin D-VDR-mediated inhibition of the transcription factor NF-κB, which binds to the promoter region of Gpx and controls its expression (12,13). Another example is the regulation of glucose-6-phosphate dehydrogenase (G6PD) expression mediated by phosphatidylinositol 3 kinase (PI3K) (14). Vitamin D signaling activates PI3K, which in turn affects the expression of G6PD. The precise mechanism by which PI3K affects G6PD expression may involve activation of downstream signaling cascades (15).

Expression of the plasma membrane calcium pump (PMCA) gene, which is responsible for regulating intracellular Ca^{2+} concentration, is strongly regulated by signaling pathways triggered by secondary messengers. In particular, protein kinase C, cyclic AMP (cAMP), and Ca^{2+} ions play critical roles in this regulatory process. The cAMP signaling pathway affects the activity of transcription factors or other regulatory proteins involved in PMCA gene expression. Ca^{2+} levels can activate various signaling cascades, including those involving transcription factors or other regulatory elements that directly control the PMCA gene (16).

Regulation of p27 expression, a key player in cell cycle regulation, is indirectly mediated by activation of transcription factors by VDR and vitamin D. Specifically, VDR has been found to enhance the expression of the transcription factor Sp1, which subsequently leads to the induction of p27 expression (17). In addition, vitamin D indirectly affects the expression of p27 through activation of Akt kinase. This in turn leads to the activation of the transcription factor AFX (also known as forkhead box protein O4, FOXO4), which plays a critical role in the induction of p27 expression (18,19). The nuclear factor erythroid 2–related factor 2 (NRF2) gene plays a critical role in cellular responses to oxidative stress, and its transcription is regulated by several transcription factors. In particular, the nongenomic vitamin D-VDR pathway modulates the activity of key transcription factors involved in the activation of NRF2 gene expression, such as NF-κB and Sp1 (11,17,20,21).

The D-VDR pathway plays a central role in regulating several genes associated with essential biological processes that contribute to hepatocyte function, chronic liver injury, and fibrosis. In particular, genes such as Gpx, PMCA, JMJD1A, LSD2, and p27 are involved in these processes (see discussion and references 54-57, 58-60, 62, 64-67 in the main text).

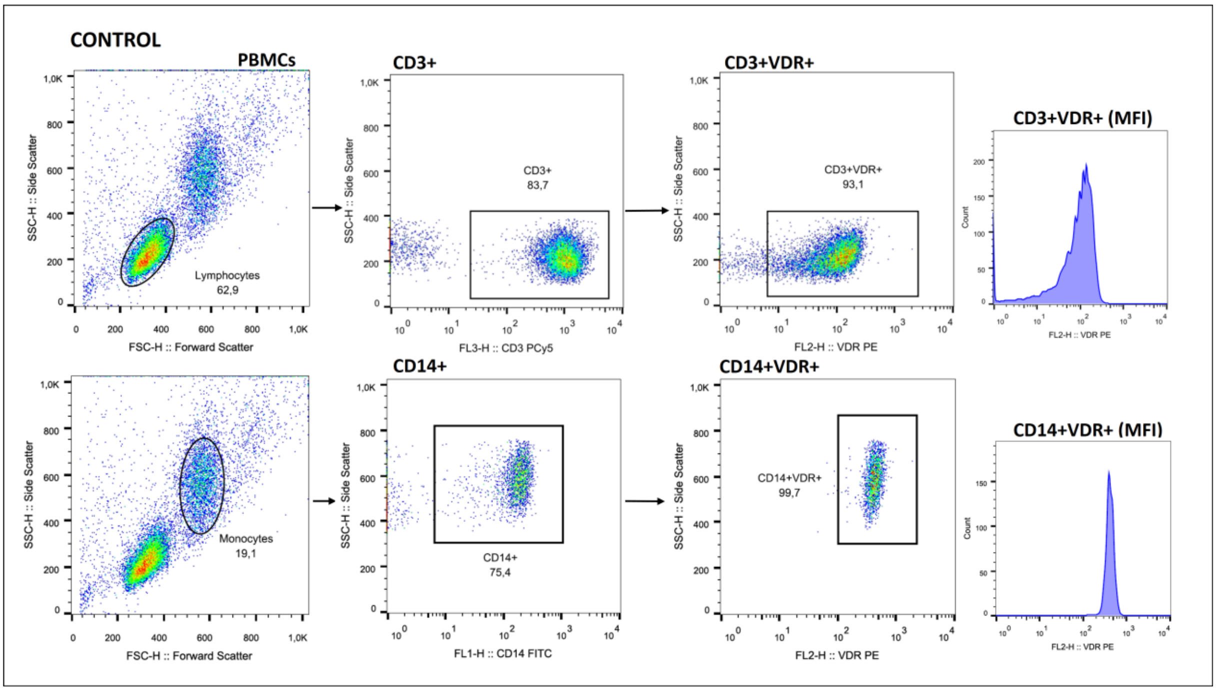

Figure S1

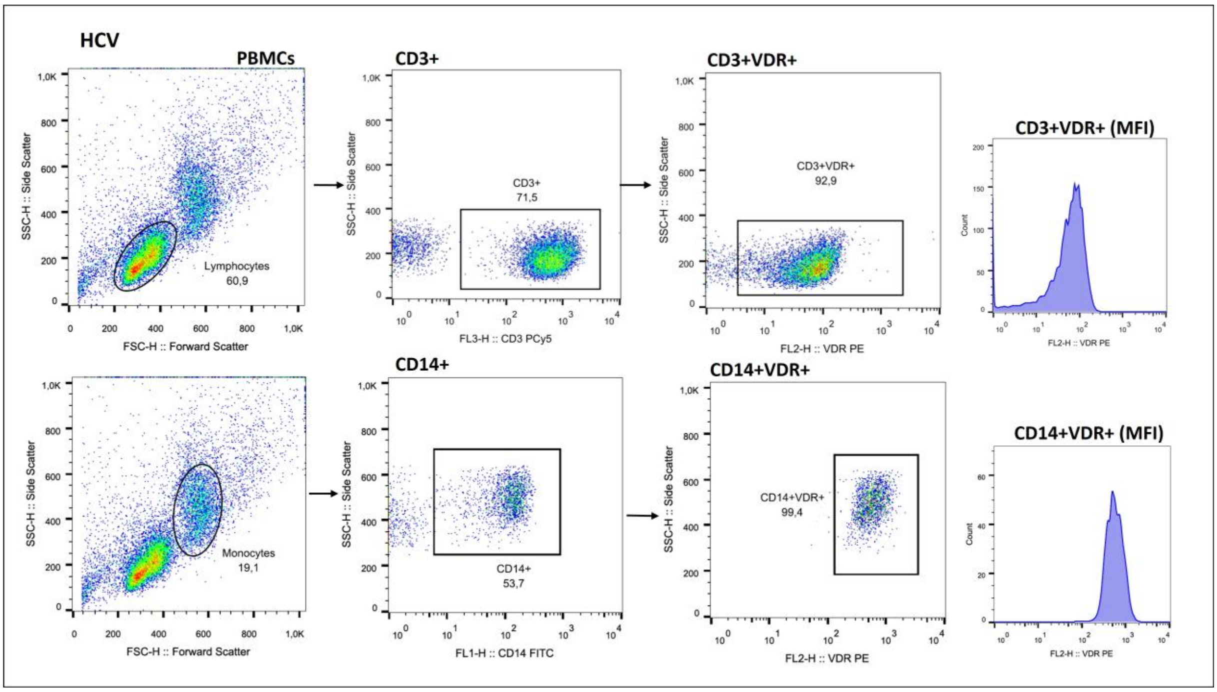

Figure S2

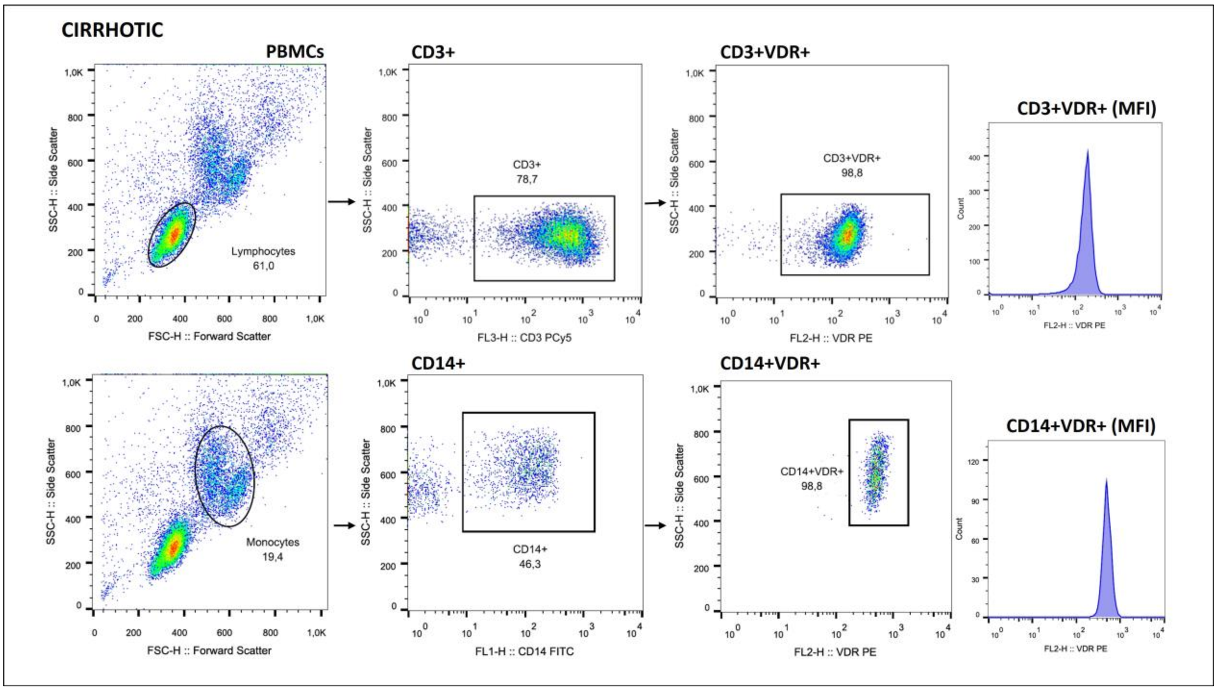

Figure S3

Figures S1-S3. Flow cytometric analysis for determination of CD3+VDR+ and CD14+VDR+ cell levels in human peripheral blood. **S1:** Representative analysis for a healthy control subject. **S2:** Representative analysis for an HCV+ patient. **S3**: Representative analysis for a cirrhotic patient. Top panel: PBMCs were gated for lymphocytes based on forward and side light scatter and analyzed for CD3 expression. Positive cells were further analyzed for VDR expression. Lower panel: PBMCs were gated for monocytes based on forward and side light scatter and analyzed for CD14 expression. Positive cells were further analyzed for VDR expression. Numbers in the dot plots indicate the percentage of gated cells expressing the relevant marker. MFI, median fluorescence intensity.

Section S4. Structure preparation and molecular dynamics (MD) simulations

Structure preparation. The crystallographic structure with PDB code 1ie9 (22)] was used to prepare the parameters for the vitamin D binding domain of the VDR. USCF Chimera (v1.11.2) (23) was used to clear the crystallographic water and remove any ligands present in the PDB files.

Design of vitamin D. The molecule of vitamin D was designed using USCF Chimera (23). Geometry optimization was performed using GAMESS software (24, 25). Density functional theory (DFT) (26, 27) with Hartree- Fock (HF) approximation was used for optimization. The atomic basis set B3LYP/6-311G was used, and the maximum convergence criterion was set to 0.0001 (27, 28). The optimized conformation of vitamin D was used to derive the atomic charges using the RESP method (29). The derivation of the charges of RESP was performed on the server RED (https://upjv.q4md-forcefieldtools.org/REDServer-Development/) (30, 31) using pyRED (30). The optimized structure of vitamin D was manually positioned in the binding site of the VDR based on the structural information of the complex crystal structure 1ie9.

MD simulations. All MD simulations were performed using AMBER14 software (32). The parameters for the different systems (wt and fokI variants in both the apo-form and in complex)

were created using the tLEap program. The ff14SB (33) force field was used to assign partial atomic charges to amino acid residues, and all histidine residues were protonated at –Nδ. The GAFF (34) force field was used to construct the parameters for the vitamin D molecule derived from the RED server for the MD simulation. A truncated octahedron box (cutoff distance of 10 Å) and periodic boundary conditions using the TIP3PBOX water model (35) were used to solvate the systems.

Minimization was performed in two steps for all systems. For the first 25000 iterations of the process, a position constraint of 100 kcal mol–1 Å–2 was imposed on the solute. This was followed by 150000 minimization steps without position constraint. The system was heated to 300 K over a period of 100 ps under constant volume conditions (NVT ensemble) using Langevin dynamic temperature scaling (36) and a collision frequency of 5 ps–1. Heating was followed by pressure equilibration for 100 ps. During both heating and pressure equilibration, a position constraint of 10 kcal mol–1 Å–2 was imposed on the solute. Finally, an unconstrained equilibration step was performed for 200 ps. The MD production simulation was performed under constant pressure and temperature conditions (NPT ensemble) for 150 ns. All bonds involving hydrogen atoms were constrained to an equilibrium distance using the SHAKE algorithm (37), while long-range electrostatic interactions were calculated using the Particle Mesh Ewald (PME) function (38).

Trajectory and cluster analysis: All analyses (e.g., rmsd, cluster calculations, and hydrogen bond interactions) were performed using the cpptraj module (39) of AMBER14. Geometric criteria for hydrogen bond (Hb) definitions were used in the analysis of interactions. The cutoff distance for donor−acceptor was 3.5 Å, and the angle cutoff for the donor−hydrogen−acceptor angle was 120°. Clustering analysis was performed using the hierarchical approach (40) during the grouping process in the different MD trajectories, using Rmsd as the distance metric (cutoff 2.5 Å).

Molecular graphics and analyses were performed using the UCSF Chimera package. Chimera was developed by the Resource for Biocomputing, Visualization, and Informatics at the University of California, San Francisco (supported by NIGMS P41-GM103311).

Table S1. Hydrogen bond (Hb) interactions in % presence during MD simulation between vitamin D and its receptor

VDR residues	Wt	fokI
Ser110	60/21	42/39/10
Tyr26	50/10	23/13
Arg106	10	18

References

1. (Ref. 6 in the main text) Christakos, S.; Dhawan, P.; Verstuyf, A.; Verlinden, L.; Carmeliet, G. Vitamin D: Metabolism, Molecular Mechanism of Action, and Pleiotropic Effects. *Physiol Rev* **2016**, *96*, 365–408, doi:10.1016/j.cgh.2019.07.060.

2. (Ref. 7 in the main text) Hanel, A.; Malmberg, H.R.; Carlberg, C. Genome-wide effects of chromatin on vitamin D signaling. *J Mol Endocrinol* **2020**, *6*4, R45–56, doi: 10.1530/JME-19-0246

3. (Ref. 8 in the main text) Bikle, D. Nonclassic actions of vitamin D. *J Clin Endocrinol Metab* **2009**, *94*, 26–34, doi:10.1210/jc.2008-1454.

4. (Ref. 9 in the main text) Masuyama, H.; Brownfield, C.M.; St-Arnaud, R.; MacDonald, P.N. Evidence for ligand-dependent intramolecular folding of the AF-2 domain in vitamin D receptor-activated transcription and coactivator interaction. *Mol Endocrinol* **1997**, *11*, 1507–17, doi:

5. (Ref. 10 in the main text) Saramäki, A.; Banwell, C.M.; Campbell, M.J.; Carlberg, C. Regulation of the human p21(waf1/cip1) gene promoter via multiple binding sites for p53 and the vitamin D3 receptor. *Nucleic Acids Res* **2006**, *34*, 543–54, doi: 10.1093/nar/gkj460.

6. (Ref. 11 in the main text) Pereira, F.; Barbáchano, A.; Singh, P.K.; Campbell, M.J.; Muñoz, A.; Larriba, M.J. Vitamin D has wide regulatory effects on histone demethylase genes. *Cell Cycle* **2012**, *11*, 1081–9, doi: 10.4161/cc.11.6.19508.

7. (Ref. 12 in the main text) Szymczak-Pajor, I.; Śliwińska, A. Analysis of Association between Vitamin D Deficiency and Insulin Resistance. *Nutrients* **2019**, *11*, 794, doi: 10.3390/nu11040794.

8. (Ref. 13 in the main text) Fleet JC. Rapid, membrane-initiated actions of 1,25 dihydroxyvitamin D: what are they and what do they mean? *J Nutr* **2004**, *134*, 3215–8, doi: 10.1093/jn/134.12.3215.

9. (Ref. 14 in the main text) Dwivedi, P.P.; Gao, X.H.; Tan, J.C.T.; Evdokiou, A.; Ferrante, A.; Morris, H.A.;May, B.K.; Hii, C.S.T.A role for the phosphatidylinositol 3-kinase--protein kinase C zeta-Sp1 pathway in the 1,25-dihydroxyvitamin D3 induction of the 25-hydroxyvitamin D3 24-hydroxylase gene in human kidney cells. *Cell Signal* **2010**, *22*, 543–52, doi: 10.1016/j.cellsig.2009.11.009.

10. (Ref. 15 in the main text) Norman, A.W. Minireview: vitamin D receptor: new assignments for an already busy receptor. *Endocrinology* **2006**, *147*, 5542–8, doi: 10.1210/en.2006-0946.

11. (Ref. 16 in the main text) Hii, C.S.; Ferrante, A. The Non-Genomic Actions of Vitamin D. *Nutrients* **2016**, *8*, 135, doi: 10.3390/nu8030135.

12. (Ref. 17 in the main text) Morgan, M.J.; Liu, Z.G. Crosstalk of reactive oxygen species and NF-κB signaling. *Cell Res* **2011**, *21*, 103–15, doi: 10.1038/cr.2010.178.

13. (Ref. 18 in the main text) Chen, Y.; Zhang, J.; Ge, X.; Du, J.; Deb, D.K.; Li, Y.C. Vitamin D receptor inhibits nuclear factor κB activation by interacting with IκB kinase β protein. *J Biol Chem* **2013**, *288*, 19450–8, doi: 10.1074/jbc.M113.467670.

14. (Ref. 19 in the main text) Wagle, A.; Jivraj, S.; Garlock, G.L.; Stapleton, S.R. Insulin regulation of glucose-6-phosphate dehydrogenase gene expression is rapamycin-sensitive and requires phosphatidylinositol 3-kinase. *J Biol Chem* **1998**, *273*, 14968–74, doi: 10.1074/jbc.273.24.14968.

15. (Ref. 20 in the main text) Doroudi, M.; Schwartz, Z.; Boyan, B.D. Membrane-mediated actions of 1,25-dihydroxy vitamin D3: a review of the roles of phospholipase A2 activating protein and Ca(2+)/calmodulin-dependent protein kinase II. *J Steroid Biochem Mol Biol* **2015**, *147*, 81–4, doi: 10.1016/j.jsbmb.2014.11.002.

16. (Ref. 21 in the main text) Kuo, T.H.; Liu, B.F.; Diglio, C.; Tsang, W. Regulation of the plasma membrane calcium pump gene expression by two signal transduction pathways. *Arch Biochem Biophys* **1993**, *305*, 428–33, doi: 10.1006/abbi.1993.1442.

17. (Ref. 22 in the main text) Huang, Y.C.; Chen, J.Y.; Hung, W.C. Vitamin D3 receptor/Sp1 complex is required for the induction of p27Kip1 expression by vitamin D3. *Oncogene* **2004**, *23*, 4856–61, doi: 10.1038/sj.onc.1207621.

18. (Ref. 23 in the main text) Stahl, M.; Dijkers, P.F.; Kops, G.J.P.L.; Lens, S.M.A.; Coffer, P.J.; Burgering, B.M.T.; Medema, R.H. The forkhead transcription factor FoxO regulates transcription of p27Kip1 and Bim in response to IL-2. *J Immunol* **2002**, *168*, 5024–31, doi: 10.4049/jimmunol.168.10.5024.

19. (Ref. 24 in the main text) Medema, R.H.; Kops, G.J.; Bos, J.L.; Burgering, B.M. AFX-like Forkhead transcription factors mediate cell-cycle regulation by Ras and PKB through p27kip1. *Nature* **2000**, *404*, 782–7, doi: 10.1038/35008115.

20. (Ref. 25 in the main text) Siswanto, F.M.; Oguro, A.; Imaoka, S. Sp1 is a substrate of Keap1 and regulates the activity of CRL4AWDR23 ubiquitin ligase toward Nrf2. *J Biol Chem* **2021**, *296*, 100704, doi: 10.1016/j.jbc.2021.100704.

21. (Ref. 26 in the main text) Gao, W.; Guo, L.; Yang, Y.; Wang, Y.; Xia, S.; Gong, H.; Zhang, B.K.; Yan, M. Dissecting the Crosstalk Between Nrf2 and NF-κB Response Pathways in Drug-Induced Toxicity. *Front Cell Dev Biol* **2022**, *9*, 809952, doi:10.3389/fcell.2021.809952.

22. (Ref. 79 in the main text) Tocchini-Valentini, G.; Rochel, N.; Wurtz, J.M.; Mitschler, A.; Moras, D. Crystal structures of the vitamin D receptor complexed to superagonist 20-epi ligands. *Proc Natl Acad Sci USA* **2001**, *98*, 5491-5496, doi: 10.1073/pnas.091018698.

23. (Ref. 74 in the main text) Pettersen, E.F. Goddard, T.D.; Huang, C.C.; Couch, G.S.; Greenblatt, D.M.; Meng, E.C.; Ferrin, T.E. UCSF Chimera-a visualization system for exploratory research and analysis. *J Comput Chem* **2004**, *25*, 1605-1612, doi: 10.1002/jcc.20084.

24. (Ref. 80 in the main text) Schmidt, M.W.; Baldridge, K.K.; Boatz, J.A.; Elbert, S.T.; Gordon, M.S.; Jensen, J.H.; Koseki, S.; Matsunaga, N.; Nguyen, K.A.; Su, S.; et al. General atomic and molecular electronic structure system. *J Comput Chem* **1993**, *14*, 1347-1363, doi:10.1002/jcc.540141112

25. (Ref. 81 in the main text) Gordon, M.S.; Schmidt, M.W. Advances in electronic structure theory: GAMESS a decade later. In: Theory and applications of computational chemistry. *Elsevier* **2005**, *Charter 41*, 1167-1189, doi: 10.1016/B978-044451719-7/50084-6.

26. (Ref. 82 in the main text) Becke, A.D. Density-functional thermochemistry. III. The role of exact exchange. *J Chem Phys* **1993**, *98*, 2155–2160, doi:10.1063/1.462066.

27. (Ref. 83 in the main text) Pople, J.A.; Gill, P.M.; Johnson, B.G. Kohn-Sham density-functional theory within a finite basis set. *Chem Phys Lett* **1992**, *199*, 557-560, doi: 10.1016/0009-2614(92)85009-Y.

28. (Ref. 84 in the main text) Hertwig, R.H.; Koch, W. On the parameterization of the local correlation functional. What is Becke-3-LYP? *Chem Phys Lett*, **1997**, *268*, 345-351, doi: 10.1016/S0009-2614(97)00207-8.

29. (Ref. 85 in the main text) Bayly, C.I.; Cieplak, P.; Cornell, W.; Kollman, P.A. A well-behaved electrostatic potential based method using charge restraints for deriving atomic charges: the RESP model. *J Phys Chem* **1993**, *97*, 10269-10280.

30. (Ref. 86 in the main text) Dupradeau, F.Y.; Pigache, A.; Zaffran, T.;Savineau, C.; Lelong, R.; Grivel, N.; Lelong, D.; Rosanski, W.; Cieplak, P. The R.E.D. tools: advances

in RESP and ESP charge derivation and force field library building. *Phys Chem Chem Phys* **2010**, *12*, 7821-7839, doi: 10.1039/c0cp00111b.

31. (Ref. 87 in the main text) Vanquelef, E.; Simon, S.; Marquant, G.; Garcia, E.; Klimerak, G.; Delepine, J.C.; Cieplak, P.; Dupradeau, F.Y. R.E.D. Server: a web service for deriving RESP and ESP charges and building force field libraries for new molecules and molecular fragments. *Nucleic Acids Res* **2011**, *39*, W511-W517, doi: 10.1093/nar/gkr288.

32. (Ref. 76 in the main text) Case, D.A., Babin, V.; Berryman, J. T.; Betz, R. M.; Cai, Q.; Cerutti, D. S.; Cheatham, T. E.; Darden, T. A.; Duke, R. E.; Gohlke, H.; Goetz, A. W.; Gusarov, S.; Homeyer, N.; Janowski, P.; Kaus, J.; Kolossváry, I.; Kovalenko, A.; Lee, T. S.; LeGrand, S.; Luchko, T.; Luo, R.; Madej, B.; Merz, K. M.; Paesani, F.; Roe, D. R.; Roitberg, A.; Sagui, C.; Salomon-Ferrer, R.; Seabra, G.; Simmerling, C. L.; Smith, W.; Swails, J.; Walker; Wang, J.; Wolf, R. M.; Wu, X.; Kollman, P. A. . Amber 14. University of California, San Francisco, **2014**. https://ambermd.org

33. (Ref. 77 in the main text) Maier, J.A.; Martinez, C.; Kasavajhala, K.; Wickstrom, L.; Hauser, K.E.; Simmerling, C. ff14SB: Improving the Accuracy of Protein Side Chain and Backbone Parameters from ff99SB. *J Chem Theory Comput* **2015**, *11*, 3696-713, doi: 10.1021/acs.jctc.5b00255.

34. (Ref. 78 in the main text) Wang, J.; Wolf, R.M.; Caldwell, J.W.; Kollman, P.A.; Case, D.A. Development and testing of a general amber force field. *J Comput Chem* **2004**, 25, 1157-74, doi: 10.1002/jcc.20035.

35. (Ref. 88 in the main text)Jorgensen, W.L.; Chandrasekhar, J.; Madura, J.D.; Impey, R.W.; Klein, M.L. Comparison of simple potential functions for simulating liquid water. *J Chem Phys* **1983**, *79*, 926-935, doi:10.1063/1.445869.

36. (Ref. 89 in the main text) Izaguirre, J.A.; Catarello, D.P.; Wozniak, J.M.; Skeel, R.D. Langevin stabilization of molecular dynamics. *J Chem Phys* **2001**, *114*, 2090-2098, doi:10.1063/1.1332996.

37. (Ref. 90 in the main text) Ryckaert, J.P.; Ciccotti, G.; Berendsen, H.J. Numerical integration of the cartesian equations of motion of a system with constraints: molecular dynamics of n-alkanes. *J Comp Phys* **1977**, *23*, 327-341.

38. (Ref. 91 in the main text) Darden, T.; York, D.; Pedersen, L. Particle mesh Ewald: An N·log (N) method for Ewald sums in large systems. *J Chem Phys* **1993**, *98*, 10089-10092, doi:10.1063/1.464397.

39. (Ref. 92 in the main text) Roe, D.R.; Cheatham, T.E. PTRAJ and CPPTRAJ: Software for Processing and Analysis of Molecular Dynamics Trajectory Data. *J Chem Theory Comput* **2013**, *9*, 3084-3095, doi: 10.1021/ct400341p.

40. (Ref. 93 in the main text) Shao, J.; Tanner, S.W.; Thompson, N.; Cheatham, T.E. Clustering Molecular Dynamics Trajectories: 1. Characterizing the Performance of Different Clustering Algorithms. *J Chem Theory Comput* **2007**, *3*, 2312-2334, doi: 10.1021/ct700119m.
